# Unexpected Associations between the Number of FRAXE Repeats in Boys and Evidence of Diabetes in Their Mothers and Maternal Grandmothers

**DOI:** 10.21926/obm.genet.2104141

**Published:** 2021-10-29

**Authors:** Jean Golding, Rosie Clark, Steven Gregory, Genette Ellis, Matthew Suderman, Yasmin Iles-Caven, Marcus E. Pembrey

**Affiliations:** 1 Centre for Academic Child Health, Bristol Medical School, University of Bristol, Oakfield House, Oakfield Grove, Bristol BS8 2BN, U.K; 2 MRC Integrative Epidemiology Unit, Population Health Sciences, Bristol Medical School, University of Bristol, Oakfield House, Oakfield Grove, Bristol BS8 2BN, U.K

**Keywords:** FRAXE, trinucleotide repeats, ALSPAC, diabetes, maternal health, maternal grandparents

## Abstract

The FRAXE section of the FMR2 gene, located on the X chromosome, contains varying numbers of trinucleotide repeats; boys with over 200 repeats tend to have mild cognitive impairments, though this is rare. Little is known, however, concerning the phenotypes of individuals with smaller numbers of repeats. Here we answer the research question as to whether the health of ancestors of boys from whom the relevant X chromosome was inherited differed in any way according to the number of FRAXE repeats. Numbers of FRAXE repeats in 5057 boys from the Avon Longitudinal Study of Parents and Children (ALSPAC) were assessed. The distribution was bimodal, with the second smaller distribution starting at 22 repeats. We tested whether possession of 22+ repeats was associated with differences in the health of mothers (who share the X chromosome) and maternal grandmothers (half of whom share it).

Female ancestors of boys with >21 repeats compared with <22 showed that maternal grandmothers (MGM) and mothers (M) had an increased risk of diabetes: MGM Type I odds ratio (OR) 2.40 [95%CI: 1.07,5.38]; MGM Type II OR 1.61 [0.96,2.70]; M OR 1.95 [0.96,3.94] using self-reported questionnaire measures. These results were confirmed from maternal medical records which revealed an increased level of diabetes [OR 2.40 (1.16,4.96)] and an increased risk of repeated glycosuria during pregnancy [OR 1.60 (1.08,2.36)]. We tested numbers of FRAXA repeats and showed no such associations, indicating that the findings were not associated with triploid repeats in general. If these findings are replicated elsewhere, there are at least three possible interpretations: (i) maternal diabetes/prediabetes results in an increased number of FRAXE repeats; (ii) women with high numbers of FRAXE repeats are at increased risk of diabetes; or (iii) some common factor, e.g. genomic instability, results in both diabetes and increased repeats.

## 1 Introduction

The FRAXE allele of the FMR2 (also known as the AFF2) gene is located at q28 on the X chromosome and contains a varying number of CCG trinucleotide repeats. The FRAXE syndrome (sometimes referred to as Fragile X E) is caused by the expansion of the CCG repeat to a full mutation [1] which occurs when the expansion is >200 repeats and becomes methylated; the rest of the distribution of repeats ranges from 0 upwards, with a mode of 15 in the UK [2, 3]. The prevalence of the full mutation at FRAXE in males has been estimated at 1 in 23,400 and is even lower for females [4]. This raises the following two questions: (i) why is there a range in the number of repeats; and (ii) do the individuals with relatively high numbers of repeats benefit in some way?

Studies comparing the number of repeats from mother to son have shown that there can be increases and decreases in the number, but this instability only occurs at levels of repeat at about 60 or more; from that point onwards, there is evidence that the rate of instability increases with the greater the number of repeats. In general, if a son has a high number of repeats at FRAXE, then his mother will almost certainly have a high number of repeats as well, although not necessarily the exact same number as her offspring. For example, in one study of over 4000 transmissions of the FRAXE repeat, changes in repeat number were remarkably uncommon; there was a bias towards expansion, but mostly the repeat number only changed by one or two repeats [2]. Therefore, if the son has a relatively high number of repeats (but <60), then it is reasonable to assume that one of the X chromosomes of his mother will also have a similar number of repeats.

The full mutation at FRAXE in males is associated with some evidence of mild cognitive impairment, behavioural problems, speech delay, reading and writing problems, poor adaptive skills, anxiety, aggression, obsessive compulsive disorder, and hyperactivity [5–10].

**Figure 1:**
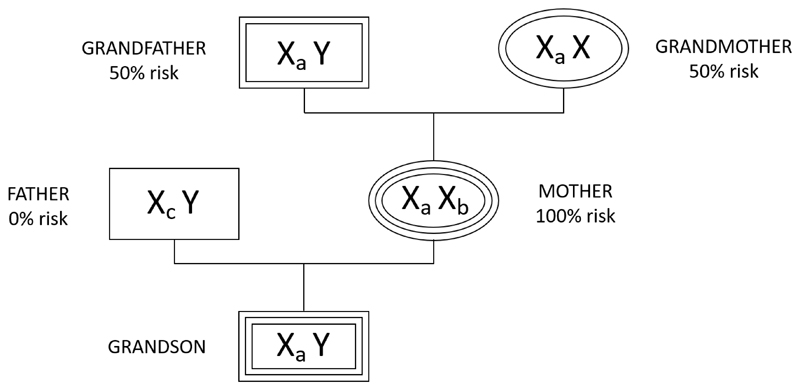
Genetic inheritance tree showing the risk of inheriting a high number of repeats at FRAXE on the X chromosome, at each generational level.

There have been few studies on phenotypes of mothers who have offspring with relatively high numbers of repeats at FRAXE. Usually, studies have been limited to cognitive phenotypes of the offspring. As indicated in the literature there is little difference between the number of repeats in the son and those in the relevant X chromosome of the mother. No studies to our knowledge have been undertaken to determine the relationship between the number of repeats on the 50% of the X chromosomes of the grandparent and the number in the grandson who inherits that chromosome (see Figure 1).

In this study, we ask the research question as to whether the maternal grandparents and mothers who transmitted the relevant X chromosome are different in their health and/or environment. We have therefore carried out a phenome scan on the mothers and maternal grandparents of boys taking part in the Avon Longitudinal Study of Parents and Children (ALSPAC), utilising the vast array of data collected from questionnaires and FRAXE repeat data obtained from DNA samples of the male offspring.

## 2 Material and Methods

### 2.1 The ALSPAC Sample

The study sample was designed to include pregnant mothers who had an expected date of delivery that was between 1st April 1991 and 31st December 1992, provided they were living in a specified area in the South-West Regional Health Authority of England [11, 12]. The original number of pregnant mothers who enrolled in the study was 14541, resulting in 13988 infants surviving to one year of age. There was an attempt to bolster the initial sample when the children in the study were around seven years of age with eligible participants who had not originally joined the study. These additional participants took the sample number to 14901 infants who had survived to one year. The majority of the data collection was from self-completion questionnaires (largely concerning psychosocial and physical environments and physical and mental health). The questionnaires were filled in by mothers, partners, the children themselves and their teachers. Further details of the study methodology [13], enrolment and response rates are available on the study website (http://bristol.ac.uk/alspac/index.html). The study website also contains details of all the available data through a fully searchable data dictionary and variable search tool (http://www.bristol.ac.uk/alspac/researchers/our-data/).

The study mothers were sent questionnaires at various stages of pregnancy, to complete in their own homes and return by post. The data collected concerned detailed structured medical histories of themselves and their parents. In addition, obstetric records were abstracted to obtain detailed information of the mother's health during pregnancy.

### 2.2 Blood Sample Collection

Consent was obtained for DNA extraction using blood from the study mothers collected during pregnancy, as well as for samples of the child's blood collected at delivery (cord blood), and for venepuncture blood taken at 43 months, 61 months, seven and nine years of age. There were some samples extracted from buccal wash when the child did not consent to venepuncture. All samples were double coded by the ALSPAC team for anonymity. This paper is concerned with the DNA extracted from bloods of 5690 boys [14].

### 2.3 DNA Amplification and Analysis

The cord blood samples collected at birth were stored in heparin at 70°C for five to eight years before DNA extraction [15]. The blood samples obtained in ALSPAC clinics from children at 43 or 61 months were stored for between one month and two years prior to DNA extraction and stored for up to three weeks for bloods taken at seven and nine years [15]. The Wessex Regional Genetics Laboratory received the samples as 250ng aliquots in 96 well plates with eight wells on each plate left empty for laboratory control; these consisted of DNA with known CCG repeat number and water controls [14]. If there were two samples stored for the same boy (e.g. cord blood and clinic sample), the clinic sample was used to minimise maternal contamination issues and to maximise genotyping efficiency, as heparin can affect PCR [14].

DNA was amplified using fluorescent PCR (using fluorescently labelled oligonucleotide primers) across the CCG FRAXE repeat. The details of the PCR reaction are given elsewhere [4, 15, 16]. After PCR, the data were analysed on 672 GENESCAN software (ABI/Perkin Elmer) and imported into GENOTYPER software (ABI/Perkin Elmer) to assign alleles [16].

### 2.4 Ethical Approval and Consent

Ethical approval for the study was obtained from the ALSPAC Ethics and Law Committee and the Local Research Ethics Committees [17]. The use of data (via questionnaires and clinics) assumed consent for the questionnaire data and required informed consent for genetic analyses obtained from participants following the recommendations of the ALSPAC Ethics and Law committee at the time. Parents completed the questionnaires in their own homes and returned them by mail to the study offices; once returned this was interpreted as giving tacit consent to involvement in the study. The details of the approvals obtained are available in full from the ethics pages of the study website (http://www.bristol.ac.uk/alspac/researchers/research-ethics/). Study participants continually have the right to withdraw their consent for elements or the entirety of the study at any time. The biological samples were collected with participants signed consent in accordance with the Human Tissue Act (2004).

### 2.5 Statistical Methodology

In the analyses, we test associations using logistic regression for binary outcomes and multiple regression for continuous outcomes. This is a hypothesis generating study and therefore we have used a P value threshold of <0.10 in an attempt to avoid type I errors. For the associations with diabetes, we have also analysed the data using the number of FRAXE trinucleotide repeats as a continuous variable to discern whether the associations shown with the binary variable is likely to be due to a general effect, or whether it applies more specifically to being in the higher bimodal levels of repeats only. Additional analyses have tested whether similar associations were found with the number of repeats at the FRAXA site.

## 3 Results

**Figure 2:**
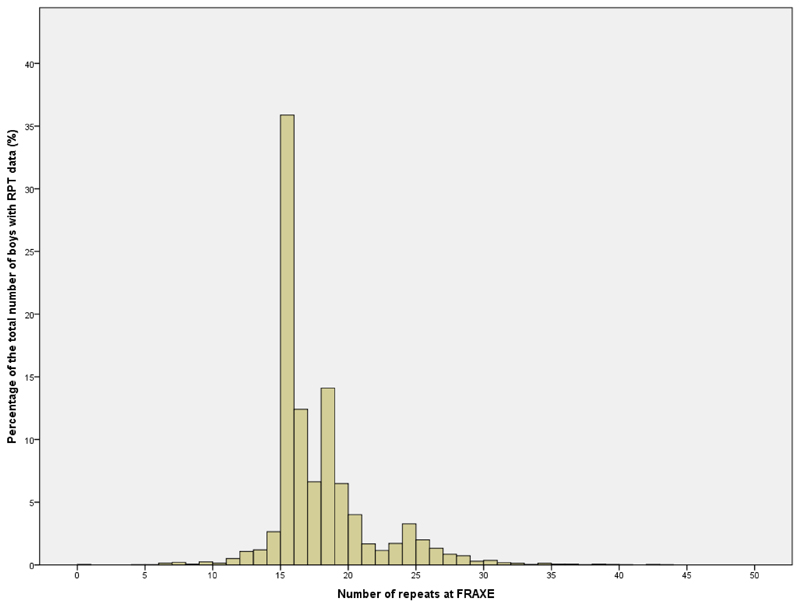
Graph of the distribution of the number of repeats at FRAXE for all boys for which valid repeat data was obtained (n = 5070).

In all, there were 5057 mother-son dyads for whom there were some data concerning the health of the mother and/or her parents. As shown in Figure 2, the distribution of the numbers of trinucleotide repeats is unusual, with a mode at 15 repeats, and indications of a bimodal distribution with a second increase starting from 22 and peaking at 24 repeats. This unusual bimodal distribution has also been found in a different UK population [2]. Based on this distribution in the present study we have selected the boys who had in excess of 21 repeats as the group with high levels of repeat and compared the health of their mothers and maternal grandparents with the rest of the population. In all, 633 (12.5%) had in excess of 21 repeats at the FRAXE site [3].

### 3.1 The Maternal Grandparents

The study mothers had reported whether or not their biological parents (the study child's maternal grandparents) had a history of particular conditions listed in the questionnaires that they completed during pregnancy. Concurrently, they reported on the various demographic characteristics of their parents. Comparison of the groups of maternal grandparents whose grandsons had >21 to those with fewer FRAXE repeats (Supplementary Table S1) showed that the grandparents did not differ in regard to the years in which they were born, or their ages when the study mother was born. There were similar proportions of non-white ethnic minority grandparents, as well as of smokers. There were slight differences, however, in the educational level of the grandparents, such that those whose grandsons had >21 FRAXE repeats had a slightly lower level of education and were slightly less likely to have had an occupation that would have been classified as professional or managerial (UK social classes I and II).

The history of medical disorders recorded for the maternal grandmothers (Table 1) encompasses 14 conditions, three of which were statistically associated with >21 repeats at P<0.05 (schizophrenia, Types I and II diabetes). The association with schizophrenia was strong (OR 4.81 [95%CI 1.70, 13.6]) but was based on only 6 cases in the high repeat group. The association with diabetes was similar at P<0.10 for each of Types I and II.

The medical history of the maternal grandfather exhibited only one out of 14 associations with P<0.10, with a reduced risk of (undefined) disability (Table 2). In contrast to the positive associations between diabetes Types I and II with >21 repeats in the maternal grandmother, the odds ratios for the maternal grandfathers were less than one, indicating a possibly reduced risk, although individually not statistically significant.

### 3.2 The Mother

For the study mother far more measures of health were available. However, there were no signs of significant differences between mothers of sons with high levels of repeats and her history of infections, surgical procedures, or psychiatric or neurological problems prior to the study pregnancy (Table 3, Table 4, Table 5 and Table 6). However, there were associations with atopic and allergic conditions such that the mother with an X chromosome with a high number of repeats was more likely to have a history of asthma and of allergy to cats, but less likely to be allergic to insect bites and stings (Table 7). Consideration of other conditions arising before the birth of the child demonstrated an excess of women with diabetes among those with a son with >21 repeats (Table 8). This excess was found whether one considered the history of a diagnosis of diabetes reported by the woman herself, or whether, during pregnancy, diabetes was recorded in the medical records and/or glycosuria had been found on two or more occasions. The only other significant finding considered an excess of psoriasis.

Finally, since there has been evidence of reproductive problems in women with high levels of FRAXA repeats, we considered the reproductive history of the women in this study. We found no indications of reproductive differences between the two groups of women – those with >21 repeats were just as likely as those with <22 repeats to have had an early or late menarche, to have become pregnant while a teenager, to have had a history of fetal loss or to have had at least three previous births (Table 9).

At the time when their sons were born, the women who had sons with >21 FRAXE repeats did not differ significantly from their contemporaries with fewer repeats in regard to their ages, ethnic background, position in the family (i.e. whether first or last born), their education level, or social class (based on their occupation). However, like their parents (Supplementary Table S1), these women had slightly lower levels of education, and were slightly less likely to have a professional or managerial occupation (Supplementary Table S2).

### 3.3 Analysis using Mean Number of Repeats

The maternal grandmothers, half of whom almost certainly had the same X chromosome as their grandson, had grandsons with higher mean levels of trinucleotide repeats if they had either Type I or Type II diabetes (mean differences 0.72 and 0.70 respectively). Their daughters, all of whom will have the same X chromosome as their son, had similarly higher mean levels of repeats if they had been diagnosed prior to the index pregnancy (mean difference 1.23 repeats), as for the sub-group in which gestational diabetes was included (mean difference 1.22). Within that sub-group, the mothers with repeated measures of glycosuria also had increased numbers of repeats (Table 10).

### 3.4 Comparison with FRAXA Repeat Number

In order to assess whether a similar pattern was found with the number of FRAXA repeats, we compared the mothers and grandmothers who had an X chromosome with the highest 10-12% of repeats (>32) repeats with those with <33. There was no sign of any positive association with diabetes (Table 11) or with any of the other disorders with higher numbers of FRAXE repeats (data not shown).

## 4 Discussion

This study is a hypothesis generator. We started with the question as to why there was such variation in the number of FRAXE repeats, with a bimodal distribution with an antimode at 22 repeats. We reasoned that the maintenance of such a variation in repeat number could be because a relatively high number of repeats may confer an advantage on the individual or merely reflect the inherent difficulty of replicating triplet repeats correctly. This study assesses the differences between three immediate ancestors of sons with high levels of repeats when compared with the rest of the sample. We have considered the mothers, their maternal grandmothers and maternal grandfathers. The mothers will almost certainly have one X chromosome with a similar number of repeats to their sons, and the grandparents will each have a 50% chance of having an X chromosome with a similar number of repeats (Figure 1).

Using our criteria for statistical significance at P<0.10, one would expect by chance that 10% of the health measures will be significantly different between high (>21) and lower numbers of repeats. We have demonstrated slightly more such P values than expected by chance for the health conditions for the maternal grandmother (expected 1.4; observed 3), fewer for the maternal grandfather (expected 1.4; observed 1), and slightly more for the mother (expected 4; observed 5). These findings would have been considered unremarkable were it not for the associations with diabetes in both the mothers and maternal grandmothers. A sensitivity analysis therefore examined other measures of diabetes in the mother and showed significant excess of diabetes diagnosed in pregnancy among mothers of the sons with >21 repeats [OR 2.40; 95% CI 1.16, 4.96; P = 0.018], as well as an excess of women who had repeated levels of glycosuria (the most common cause of which is diabetes) [OR 1.60; 95% CI 1.08, 2.36; P = 0.020].

We examined the possibility that the association might be similar for other trinucleotide repeats by testing higher levels of FRAXA repeats for all outcomes found associated statistically significantly with FRAXE. None showed any associations that were similar to those found for FRAXE.

### 4.1 Strengths and Limitations

The strengths of the study lie in the following: (a) It is nested within a population birth cohort; unlike other studies of family members, this study is not contingent on the presence of a family member with Fragile XE. (b) Data were collected from the mothers concerning the health histories of themselves and their parents during pregnancy, prior to the birth of their sons. (c) Assays to determine the number of FRAXE repeats were carried out without knowledge of the health and development of the study mother or her parents. (d) Comparisons of results using a binary measure of high numbers of repeats, with the results of the mean numbers using linear regression indicated a similar pattern of associations.

Limitations concern: (i) the fact that we do not know which of the maternal grandparents had an X chromosome with >21 repeats. (ii) The features of medical history were obtained from the mother, and rarely from medical records. However, medical records were also used to obtain details of pregnancy for a subsample of women, and a similar association was found for a diagnosis of diabetes noted in medical records. (iii) We did not allow for confounders as we did not find any biological or environmental features that distinguished the two groups of mothers or grandmothers, but there may have been some features that should have been taken into account.

## 5 Conclusions

This is a study that started with a blank page in regard to what we would be likely to find, other than the question as to why the lengths of the repeat sequence in the promoter region of the FMR2 gene varied to such an extent. Our findings of increased risk of diabetes in the women who were direct ancestors of the boys with >21 repeats is intriguing, with results confirmed by analyses of mean trinucleotide repeats. If confirmed elsewhere these results may provide a clue concerning the development of diabetes among women.

## Supplementary Material

Supplementary material

## Figures and Tables

**Table 1:** The association of the medical history of the maternal grandmother with the number of FRAXE repeats.

Condition	>21 repeats **N (%)**	<22 repeats **N (%)**	OR [95% CI]	P
N	539 (100)	4117 (100)		
Diabetes Type I	8 (1.5)	24 (0.6)	2.40 [1.07, 5.38]	0.033
Diabetes Type II	18 (3.3)	81 (2.1)	1.61 [0.96, 2.70]	0.073
Coronary heart disease	22 (4.1)	112 (2.9)	1.42 [0.89, 2.26]	0.140
A stroke	9 (1.7)	79 (2.1)	0.81 [0.40, 1.63]	0.555
Hypertension	124 (23.0)	783 (20.3)	1.17 [0.94, 1.45]	0.151
Rheumatism	81 (15.0)	529 (13.7)	1.11 [0.86, 1.43]	0.416
Arthritis	132 (24.5)	901 (23.4)	1.06 [0.86, 1.31]	0.573
Chronic bronchitis	13 (2.4)	137 (3.6)	0.67 [0.38, 1.19]	0.173
Breast cancer	20 (3.7)	135 (3.5)	1.06 [0.66, 1.71]	0.808
Other cancer	39 (7.2)	236 (6.1)	1.20 [0.84, 1.70]	0.320
Alcohol problem	14 (2.6)	78 (2.0)	1.29 [0.73, 2.30]	0.386
Schizophrenia	6 (1.1)	9 (0.2)	4.81 [1.70, 13.6]	0.003
Depression	109 (20.2)	790 (20.5)	0.98 [0.79, 1.23]	0.877
Disabled	32 (6.0)	239 (6.3)	0.95 [0.65, 1.39]	0.795

**Table 2:** The association of the medical history of the maternal grandfather with the number of FRAXE repeats.

Condition	>21 repeats **N (%)**	<22 repeats **N (%)**	OR [95% CI]	P
N	539 (100)	3852 (100)		
Diabetes Type I	<5 (0.4)	43 (1.1)	0.33 [0.08, 1.37]	0.126
Diabetes Type II	9 (1.7)	106 (2.8)	0.60 [0.30, 1.19]	0.145
Coronary heart disease	42 (7.8)	387 (10.0)	0.76 [0.54, 1.05]	0.100
Stroke	15 (2.8)	128 (3.3)	0.83 [0.48, 1.43]	0.509
Hypertension	73 (13.5)	612 (15.9)	0.83 [0.64, 1.08]	0.161
Rheumatism	38 (7.1)	261 (6.8)	1.04 [0.73, 1.49]	0.813
Arthritis	55 (10.2)	407 (10.6)	0.96 [0.71, 1.29]	0.798
Chronic bronchitis	26 (4.8)	174 (4.5)	1.07 [0.70, 1.63]	0.749
Prostate cancer	5 (0.9)	26 (0.7)	1.38 [0.53, 3.60]	0.513
Other cancer	28 (5.2)	201 (5.2)	1.00 [0.66, 1.49]	0.982
Alcohol problem	37 (6.9)	201 (5.2)	1.34 [0.93, 1.92]	0.115
Schizophrenia	<5 (0.2)	18 (0.5)	0.40 [0.05, 2.97]	0.368
Depression	35 (6.5)	310 (8.0)	0.79 [0.55, 1.14]	0.210
Disabled	28 (5.3)	290 (7.7)	0.66 [0.45, 0.99]	0.044

**Table 3:** The association of the maternal history of common infections with the number of FRAXE repeats.

Condition	>21 repeats N (%)	<22 repeats N (%)	OR [95% CI]	P
Measles	385 (71.6)	2756 (71.8)	0.99 [0.81, 1.21]	0.919
Mumps	275 (51.1)	2103 (54.8)	0.86 [0.72, 1.03]	0.112
Chicken pox	463 (86.1)	3322 (86.5)	0.96 [0.74, 1.25]	0.775
Pertussis	53 (9.9)	440 (11.5)	0.84 [0.63, 1.14]	0.270
Herpes simplex	239 (44.4)	1652 (43.0)	1.06 [0.88, 1.27]	0.538
Genital herpes	13 (2.4)	74 (1.9)	1.26 [0.69, 2.29]	0.447
Urinary infection	278 (51.7)	2099 (54.7)	0.89 [0.74, 1.06]	0.193

**Table 4:** The association of the maternal anthropometry pre-pregnancy with the number of FRAXE repeats.

Pre-pregnancy measurement	>21 repeats Mean (SD)	<22 repeats Mean (SD)	MD [95% CI]	N	P
Hip circumference (cm)	36.33 (2.66)	36.45 (2.53)	-0.13 [-0.43, +0.18]	2514	0.414
Waist circumference (cm)	26.92 (2.90)	26.96 (2.66)	-0.04 [-0.35, +0.26]	2807	0.787
Bust circumference (cm)	35.24 (2.21)	35.42 (2.19)	-0.18 [-0.40, +0.04]	3572	0.110
Weight (kg)	62.15 (10.80)	61.73 (10.88)	+0.42 [-0.59, +1.43]	4125	0.411
Height (in)	64.46 (2.76)	64.62 (2.63)	-0.16 [-0.40, +0.08]	4305	0.199
BMI	23.19 (3.88)	22.91 (3.81)	+0.28 [-0.08, +0.63]	4075	0.129

BMI = body mass index; MD = mean difference; SD = standard deviation

**Table 5:** The association of the maternal history of common surgical procedures with the number of FRAXE repeats.

Procedure	>21 repeats	<22 repeats	OR [95% CI]	P
	N (%)	N (%)		
Tonsillectomy	110 (20.5)	855 (22.4)	0.89 [0.71, 1.12]	0.320
Adenoidectomy	56 (10.5)	472 (12.5)	0.82 [0.61, 1.10]	0.185
Appendicectomy	35 (6.5)	304 (8.0)	0.80 [0.56, 1.16]	0.240
Squint repair	14 (2.6)	78 (2.1)	1.28 [0.72, 2.28]	0.396

**Table 6:** The association of the maternal history of common neurological and psychiatric problems pre-pregnancy with the number of FRAXE repeats.

Condition	>21 repeats	<22 repeats	OR [95% CI]	P
	N (%)	N (%)		
Bulimia	12 (2.2)	81 (2.1)	1.06 [0.57, 1.96]	0.852
Anorexia nervosa	12 (2.2)	81 (2.1)	1.06 [0.57, 1.96]	0.852
Severe depression	48 (9.0)	312 (8.2)	1.17 [0.84, 1.64]	0.696
Other psychiatric condition	9 (1.7)	87 (2.3)	0.73 [0.37, 1.47]	0.383
Febrile convulsions	10 (1.9)	79 (2.1)	0.90 [0.46, 1.75]	0.763
Migraine	82 (15.6)	563 (15.0)	1.01 [0.78, 1.31]	0.718

**Table 7:** The association of maternal markers of allergies and atopic disease with the number of FRAXE repeats.

Condition*	>21 repeats N (%)	<22 repeats N (%)	OR [95% CI]	P
Asthma recently	49 (9.2)	260 (6.8)	1.38 [1.00, 1.96]	0.063
Eczema	55 (10.4)	429 (11.3)	0.93 [0.69, 1.25]	0.957
Hay fever	114 (21.6)	698 (18.6)	1.18 [0.94, 1.47]	0.282
Allergic to cats	63 (11.8)	347 (9.1)	1.33 [1.00, 1.77]	0.048
Allergic to pollen	99 (18.5)	631 (16.6)	1.15 [0.91, 1.45]	0.257
Allergic to dust	81 (15.2)	502 (13.2)	1.18 [0.91, 1.52]	0.210
Allergic to insect bites/stings	19 (3.6)	207 (5.4)	0.64 [0.40, 1.04]	0.069
Any allergies	227 (43.2)	1652 (43.5)	0.99 [0.82, 1.19]	0.899

* Compared to never had for Asthma, Eczema, Hay Fever

**Table 8:** The association of the history of other conditions of mother (all conditions concern ever had except when indicated that the condition was recent*).

Condition	>21 repeats N (%)	<22 repeats N (%)	OR [95% CI]	P
Indigestion*	243 (45.5)	1685 (44.9)	1.06 [0.86, 1.32]	0.603
Stomach ulcer	9 (1.7)	57 (1.5)	1.13 [0.56, 2.30]	0.734
Pelvic inflammatory disease	17 (3.2)	93 (2.4)	1.32 [0.78, 2.23]	0.305
Kidney disease	25 (4.6)	161 (4.2)	1.12 [0.72, 1.72]	0.621
Varicose veins*	50 (9.4)	395 (10.4)	0.89 [0.65, 1.21]	0.405
Haemorrhoids*	92 (17.3)	638 (16.7)	1.04 [0.81, 1.33]	0.819
Diabetes ever	10 (1.9)	37 (1.0)	1.95 [0.96, 3.94]	0.064
Diabetes noted in pregnancy	10 (2.7)	29 (1.1)	2.40 [1.16, 4.96]	0.018
Glycosuria 2+ on 2+ occasions	32 (5.5)	145 (3.5)	1.60 [1.08, 2.36]	0.020
Hypertension	84 (15.8)	540 (14.3)	1.13 [0.88, 1.45]	0.336
Back pain*	128 (23.9)	943 (24.7)	0.95 [0.76, 1.19]	0.659
Rheumatism*	13 (2.4)	54 (1.4)	1.73 [0.94, 3.18]	0.160
Arthritis*	17 (3.2)	65 (1.7)	1.90 [1.10, 3.26]	0.049
Psoriasis*	16 (3.0)	77 (2.1)	1.49 [0.86, 2.58]	0.089

**Table 9:** The association of the reproductive history of mother prior to the conception of her son with the number of FRAXE repeats.

Reproductive history	>21 repeats N (%)	<22 repeats N (%)	OR [95% CI]	P
<12 at menarche	82 (15.2)	688 (17.9)	0.83 [0.64, 1.06]	0.131
Ever used contraceptive pill	509 (94.6)	3600 (93.7)	1.18 [0.79, 1.75]	0.413
Ever pregnant before	382 (68.2)	2693 (67.2)	1.05 [0.87, 1.27]	0.629
<20 at 1^st^ pregnancy	94 (16.8)	689 (17.2)	0.97 [0.75, 1.26]	0.787
Ever miscarried	128 (22.9)	857 (21.6)	1.08 [0.87, 1.33]	0.482
Ever terminated	75 (13.5)	554 (14.0)	0.96 [0.74, 1.25]	0.762
Had had a stillbirth	7 (1.3)	39 (1.0)	1.28 [0.57, 2.87]	0.554
Parity 3+ at index pregnancy	31 (5.6)	225 (5.7)	1.03 [0.69, 1.54]	0.433

**Table 10:** Comparison of the mean number of FRAXE trinucleotide repeats of boys in relation to the measures of diabetes in their maternal ancestors.

History	n	Mean difference	95% confidence limits	P
*Maternal Grandmother (MGM)*				
Type I diabetes	32	+0.72	-0.65, +2.09	0.305
Type II diabetes	99	+0.70	-0.08, +1.49	0.079
*Mother (M)*				
Diabetes prior to study pregnancy	47	+1.23	+0.09, 2.36	0.034
Diabetes in pregnancy	39	+1.22	-0.02, +2.46	0.053
Glycosuria on 2+ occasions in pregnancy	177	+0.84	+0.25, 1.43	0.006

**Table 11:** The association of the maternal and grandmaternal history of diabetes of repeat glycosuria with the number of FRAXA repeats, comparing >32 with <33.

History	n	Odds ratio	95% confidence Limits	P
*Maternal Grandmother (MGM)*				
Type I diabetes	32	0.52	[0.12, 2.19]	0.374
Type II diabetes	99	0.90	[0.47, 1.74]	0.756
*Mother (M)*				
Diabetes prior to study pregnancy	47	0.35	[0.08, 1.45]	0.147
Diabetes in pregnancy	39	0.67	[0.21, 2.19]	0.508
Glycosuria on 2+ occasions in pregnancy	177	0.92	[0.72,1.17]	0.120
