# Supplementary material for: Unexpected Associations between the Number of FRAXE Repeats in Boys and Evidence of Diabetes in Their Mothers and Maternal Grandmothers
Source: OBM Genet. Author manuscript; Available in PMC 2022 Apr 29. (PMC7612666; doi:10.21926/obm.genet.2104141)
Supplement: Supplementary material [file EMS144007-supplement-Supplementary_material.pdf]

Supplementary Material

Supplementary Table 1. Characteristics of the grandparents.

Characteristic	MGM >21 repeats	MGM <22 repeats	MGM P	MGF >21 repeats	MGF <22 repeats	MGF P
	**Mean (SD)**	**Mean (SD)**		**Mean (SD)**	**Mean (SD)**	
Year of birth of grandparent	1935.1 (8.2)	1934.9 (8.5)	0.657	1931.7 (9.0)	1931.7 (9.1)	0.884
Age at birth of mother	27.1 (5.8)	27.0 (5.8)	0.732	30.4 (7.0)	30.0 (6.6)	0.214
	**N (%)**	**N (%)**	**P**	**N (%)**	**N (%)**	**P**
Social class* I&II	73 (26.0)	596 (28.2)	0.452	136 (32.7)	1102 (35.9)	0.200
Non-white	12 (2.2)	79 (2.1)	0.791	13 (2.4)	87 (2.3)	0.827
Education level >O-level equivalent	137 (34.7)	1062 (36.4)	0.544	139 (37.0)	1070 (38.8)	0.504
Smoker	308 (56.9)	2116 (55.3)	0.480	402 (75.7)	2840 (75.4)	0.859

*based on the occupation of the grandparent. MGM = maternal grandmother; MGF = maternal grandfather.

Supplementary Table 2. Demographic characteristics of the study mother.

Characteristic of mother	>21 repeats N (%)	<22 repeats N (%)	OR [95% CI]	P
Born preterm	45 (8.6)	264 (7.1)	1.23 [0.89, 1.72]	0.258
Non-white	10 (1.9)	95 (2.5)	0.75 [0.39, 1.44]	0.381
Born in Avon	270 (55.1)	1946 (54.8)	1.01 [0.84, 1.22]	0.895
First-born	156 (28.4)	1252 (32.4)	0.82 [0.63, 1.08]	0.153
Last-born	199 (36.2)	1332 (34.5)	1.15 [0.90, 1.48]	0.269
Education >O-level	181 (33.1)	1482 (38.5)	0.83 [0.64, 1.08]	0.164
Social class I or II	150 (31.5)	1133 (32.6)	0.89 [0.68, 1.17]	0.417
	**Mean (SD)**	**Mean (SD)**	**MD [95% CI]**	**P**
Year of birth	1962.9 (4.7)	1962.7 (4.9)	+0.20 [-0.23, +0.62]	0.362
Age at LMP	27.6 (4.7)	27.8 (4.9)	-0.21 [-0.63, +0.21]	0.329
